# The mechanisms of nature-based therapy on depression, anxiety, stress, and life satisfaction: examining mindfulness in a two-wave mediation model

**DOI:** 10.3389/fpsyg.2023.1330207

**Published:** 2023-12-22

**Authors:** Minjung Kang, Yeji Yang, Hyunjin Kim, Songhie Jung, Hye-Young Jin, Kee-Hong Choi

**Affiliations:** ^1^School of Psychology, Korea University, Seoul, Republic of Korea; ^2^KU Mind Health Institute, Korea University, Seoul, Republic of Korea; ^3^Gardens and Education Research Division, Korea National Arboretum, Pocheon, Republic of Korea

**Keywords:** nature-based therapy, psychological mechanism, mindfulness, depression, anxiety, stress, life satisfaction

## Abstract

**Background:**

Nature-based therapy (NBT), which centers around engaging in activities within natural surroundings, has consistently demonstrated therapeutic benefits for mental health. While NBT highlights the potential of nature as a therapeutic resource for promoting mental health, there is limited knowledge regarding its underlying mechanisms.

**Methods:**

Two hundred seventy-six Korean participants (204 women, mean age = 54.99 ± 23.25 years) participated in a 30-session gardening program held twice weekly for 15 weeks. Structural equation modeling with a two-wave autoregressive cross-lagged model was used to investigate the mediating effects of mindfulness.

**Results:**

NBT significantly improved the mean scores of all psychological variables. The mediation model was partially confirmed, with mindfulness at post-intervention (T2) mediating the relationship between baseline (T1) depression and anxiety and post-intervention (T2) life satisfaction. However, no significant indirect effect was observed between the path from stress (T1) to life satisfaction (T2).

**Conclusion:**

Mindfulness is a crucial component for improving mental health outcomes. This study underscores the need to prioritize and emphasize mindfulness practices in NBT.

## 1 Introduction

The emergence of the Covid-19 pandemic and ongoing concerns have significantly impacted global mental health, leading to increased rates of depression, anxiety, and stress (Schafer et al., 2022; Mahmud et al., 2023). In South Korea, the pandemic has been linked to increased depression, perceived stress, and suicidal plans along with diminished quality of life (Park et al., 2021; Jeong et al., 2022). The pandemic’s impact has been particularly profound among socially vulnerable populations, such as those facing barriers to healthcare and economic resources, as well as people with pre-existing health conditions or disabilities (Flaskerud and Winslow, 1998; Mechanic and Tanner, 2007; Calderón-Larrañaga et al., 2020; Li et al., 2023). During the Covid-19 pandemic, elderly with chronic illnesses faced economic hardships, which intensified their susceptibility to mental health challenges through restricted access to community services (McArthur et al., 2021; García-Prado et al., 2022). Additionally, the pandemic has introduced unique mental health stressors in younger individuals, with education disruptions and limited social contact leading to increased anxiety (Singh et al., 2020; Kauhanen et al., 2023). People with physical and mental disabilities have faced increased health vulnerabilities, reduced social connections, and limited healthcare services, leading to heightened social isolation (Kim et al., 2020; Dalise et al., 2021). Research indicates that the impact of mental health was more detrimental for the vulnerable population, with those suffering from depression or anxiety reporting greater levels of distress due to less adaptive coping strategies (Calderón-Larrañaga et al., 2020; Diaz et al., 2021; Solé et al., 2021). Hence, more attention and necessary interventions are essential to address these disparities in mental health and well-being. These challenges have prompted a call for innovative psychosocial interventions that extend beyond traditional clinical settings. Policymakers and mental health experts call for a novel, community-oriented treatment to address enduring impacts of the pandemic (Moreno et al., 2020). At the same time, the natural synergy between humans and nature is guiding a growing emphasis on nature-based therapies aimed at enhancing psychological well-being and development.

Nature exposure is often positively linked to both physical and psychological well-being (Mitchell and Popham, 2008; Bowler et al., 2010). Nature-based therapy (NBT), alternatively referred to as natural therapy, nature-assisted therapy, or green care, is a therapeutic approach centered around engaging in activities within natural surroundings (Corazon et al., 2010). NBT encompasses various forms, including horticulture, therapeutic gardening, and interventions in natural environments like wilderness therapy and forest bathing (Annerstedt and Währborg, 2011; Hansen et al., 2017). Various interventions have shown promising results in reducing depression, anxiety, and stress, and improving cognitive functions and overall well-being (Maas et al., 2009; Beyer et al., 2014; Stigsdotter et al., 2018; Ainamani et al., 2021; Yang et al., 2022). These findings algin with observations from South Korea. For instance, the systematic review on the health benefits of forest therapy showed that participants experienced improved mindfulness and physical activity levels (Park et al., 2021). In addition, middle-aged Korean women who participated in horticultural therapy reported reductions in depression and anxiety symptoms, along with increased self-identity (Kim and Park, 2018). Nature-based interventions have also been effective for diverse populations, including individuals with schizophrenia spectrum disorders, veterans, and college students, highlighting the nature’s potential as a therapeutic resource for promoting psychological well-being in both clinical and non-clinical groups (Kam and Siu, 2010; McMahan and Estes, 2015; Stowell et al., 2018).

Although careful consideration is needed in interpreting the connection between NBT and enhanced health outcomes (Soga et al., 2017), evidence suggests that NBT utilizes mindfulness (Cimprich and Ronis, 2003; Howell et al., 2011; Van Gordon et al., 2018). Mindfulness is a state of consciousness or awareness that emerges from actively focusing on the present moment in an accepting and nonjudgmental manner (Kabat-Zinn, 2009). Therapeutic gardening activities align with the elements of mindfulness incorporating their inherent connection to nature and the ability to promote attention to the present (Cimprich and Ronis, 2003; Mori et al., 2021). A meta-analysis on nature-based mindfulness revealed that interventions elicit positive psychological effects, with enhanced benefits when performed in natural environments (Djernis et al., 2019). This is in line with Kaplan’s (1995) attention restoration theory, which posits that nature allows individuals to experience restorative effects by effortlessly diverting attention.

Given the positive influence of mindfulness on psychological well-being, the current study focused on pivotal psychological variables including depression, anxiety, stress, and life satisfaction, which are closely linked to mindfulness. Depression is characterized by enduring feelings of sadness, diminished interest or pleasure in activities, alterations in appetite or weight, sleep disruptions, fatigue, and thoughts of worthlessness or death (American Psychiatric Association, 2022). Nature’s impact on alleviating depressive symptoms is well documented in various studies (Chu et al., 2019; Yeon et al., 2021; Grassini, 2022). Moreover, studies have demonstrated a significant inverse relationship between mindfulness and depression (Baer, 2003; Grossman et al., 2004; Christopher and Gilbert, 2010). Studies have shown that mindfulness-based interventions (MBI) reduce depressive symptoms and prevent relapse in individuals with a history of depression (Hofmann et al., 2010; Segal et al., 2018). The findings of a multi-center randomized controlled trial demonstrated that individuals with depression who engaged in a nature-based group alongside standard care exhibited significant reductions in psychological distress and improvements in restoration, indicating that the restorative experiences provided by nature played a mediating role in diminishing depressive symptoms (Hyvönen et al., 2023).

Anxiety refers to persistent feelings of worry, fear, and unease involving physiological symptoms such as increased heart rate and restlessness (American Psychiatric Association, 2022). Greater time spent in nature is associated with positive mental health outcomes, including reduced anxiety (Bowler et al., 2010; Bratman et al., 2015; Keenan et al., 2021; Lackey et al., 2021). In addition to natural environments, meta-analyses indicated that MBI was effective in reducing anxiety symptoms in both healthy individuals and clinical samples (Hofmann et al., 2010; Khoury et al., 2015; Jimenez et al., 2021). Furthermore, a meta-analysis on the effects of Shinrin-Yoku, also known as forest bathing, which considers mindfulness as a fundamental element, has been effective in reducing anxiety (Kotera et al., 2022).

Stress refers to a physiological and psychological response to perceived threats or demands that exceed an individual’s coping abilities, resulting in a state of tension and strain (Cohen et al., 1995). MBI was originally developed for stress reduction (Kabat-Zinn, 1982). Studies have consistently demonstrated an inverse relationship between mindfulness and stress across various populations (Foster, 2007; Nezlek et al., 2016; Querstret et al., 2020; Sousa et al., 2021). In a meta-analysis, mindfulness training was found to significantly reduce stress reactivity, including physiological markers (Pascoe et al., 2017; Querstret et al., 2020). Additionally, one study revealed that increased mindfulness training played a mediating role in increasing positive emotions and reducing stress and cortisol levels (Sousa et al., 2021). Another study indicated that individuals with higher levels of mindfulness tended to perceive challenging situations as less stressful and threatening and were more likely to utilize adaptive coping strategies (Weinstein et al., 2009). In relation to nature, a randomized controlled trial investigating the effectiveness of NBT in treating individuals with a stress-related illness found that both the NBT group and the cognitive-behavioral therapy control group showed significant improvement in stress levels at treatment completion (Stigsdotter et al., 2018).

Life satisfaction refers to an individual’s overall evaluation of their life (Diener et al., 1999). Nature’s positive effect on well-being is well documented (Bowler et al., 2010; Koay and Dillon, 2020; Sadowski et al., 2022). Even a brief exposure to natural environments seems to increase emotional well-being (McMahan and Estes, 2015). Owing to its cost effectiveness and promising results on promoting life satisfaction and happiness, several countries have adopted NBT as a public health policy (Chan et al., 2017; Gagliardi and Piccinini, 2019; Koay and Dillon, 2020; Pretty and Barton, 2020). Additionally, studies indicate that individuals with higher levels of mindfulness tend to report greater life satisfaction (Keng et al., 2011; Davis and Hayes, 2012; Schirda et al., 2015; Bajaj and Pande, 2016). Garland et al. (2015) proposed a model that posited mindfulness enhances life satisfaction by broadening one’s awareness and promoting positive emotion regulation, leading to a greater sense of well-being.

While the effects of NBT and mindfulness interventions in reducing negative affect and increasing life satisfaction are well documented in numerous studies, few studies have focused on examining mindfulness as a mediator. Dehghan et al. (2020) investigated the relationship between stress, quality of life, and mindfulness in patients with cancer. Findings revealed that mindfulness played a mediating role in reducing perceived stress, which resulted in increased quality of life. Mindfulness also partially mediated the positive outcome of an MBI; participants who had higher levels of mindfulness experienced reductions in perceived stress, anxiety, and depressive symptoms, leading to enhanced overall well-being and quality of life (Nyklícek and Kuijpers, 2008). Huynh and Torquati (2019) suggested that both connection to nature and mindfulness play important roles in promoting psychological well-being. Specifically, they found that mindfulness mediated the relationship between nature connection and various psychological outcomes, including life satisfaction, positive affect, and vitality. Hence, enhanced mindfulness could mediate the positive effects of psychological well-being.

Research in the field of NBT often lacks investigations into the longitudinal mediators that contribute to observed therapeutic effects. Therefore, this study aimed to explore how engagement in nature-based activities influences mindfulness over time, and how mindfulness, in turn, mediates the relationship between life satisfaction and depression, anxiety, and stress. This study aimed to offer insights regarding the function of mindfulness as a key mechanism in NBT, contributing to a deeper understanding of the therapeutic effects of nature on psychological well-being in the context of Covid-19. The following hypotheses were proposed for the two-wave autoregressive cross-lagged mediation model.

*H1:* Mindfulness at Time 2 (T2) mediates the relationship between depression at Time 1 (T1) and life satisfaction (T2).

*H2:* Mindfulness (T2) mediates the relationship between anxiety (T1) and life satisfaction (T2).

*H3:* Mindfulness (T2) mediates the relationship between stress (T1) and life satisfaction (T2).

## 2 Method

### 2.1 Participants

In total, 276 participants (204 women, 72 men, mean age = 54.99 ± 23.25 years) were recruited from 11 different institutions serving vulnerable populations nationwide in Korea from February to April 2022. Participants were recruited through advertisements distributed to four senior welfare centers (*n* = 103, 37.3%), two medical centers (*n* = 59, 21.4%), a local university (*n* = 30, 10.9%), a botanic garden (*n* = 28, 10.1%), a special school (*n* = 21, 7.6%), a community center (*n* = 20, 7.2%), and a mental health center (*n* = 15, 5.4%). Participants were (1) individuals aged 13 or older, and (2) identified as belonging to one or more vulnerable groups based on the criteria developed to investigate Covid-19’s impact on such populations (Li et al., 2023). The criteria include: (a) Cognitive or communicative vulnerability (e.g., poor mental health conditions, decisionally impaired) (b) Institutional or deferential vulnerability (e.g., prisoners, or children/students) (c) Health vulnerability (e.g., disabled, terminally ill, older people) (d) Economic vulnerability (e.g., dependent or impoverished subjects) (e) Social vulnerability (e.g., minorities). Vulnerable groups are not mutually exclusive and may overlap. Individuals who had impediments that would preclude program participation, such as mobility constraints, communication difficulties, or severe mental disorder (e.g., schizophrenia) were excluded. The assessment criteria were examined through diagnostic interviews and validated mental health screening tools. The study obtained approval from the Korea University Institutional Review Board (Protocol No. KUIRB-2022-0218-03, 05/04/2022). All participants gave written informed consent, and for those under 18 years old, written consent was obtained from their parent or legal guardian.

### 2.2 Intervention

This study is part of a larger study investigating the effectiveness and feasibility of NBT in addressing psychological distress within the community amid the Covid-19 pandemic. In the larger study, the NBT group exhibited moderate to large effect size in alleviating psychological distress and improving well-being compared to the control group (Yang et al., manuscript submitted). The intervention was implemented across 11 institutes, consisting of 30 sessions conducted over 15 weeks from April to July 2022. Sessions were held twice weekly and each lasted for 2 h. Contents primarily involved gardening-related activities (85%), supplemented by leisure activities, such as flower arrangements, yoga, and picnics (15%). Each institution had their own dedicated gardens for participants’ use in the therapeutic gardening activities, with the program structured as a group therapy. Group size varied, with the smallest being 14 participants and the largest 37 participants. For optimal engagement, subgroups of 6–10 people were occasionally formed within a larger group, yet all participants engaged in uniform program activities. The program curriculum, adaptable to local and weather conditions, followed the manual suggested by the Korea National Arboretum and was reviewed by clinical psychologists and horticultural therapy experts. Details of the program’s framework are presented in Table 1.

**Table 1:** The contents of the therapeutic gardening program.

Session	Activity	Session	Activity
1	Introduction	16	Potting 2: setting up mini garden
2	Preparing gardening	17	Event 3: making potpourri
3	Setting up garden 1: making bed	18	Designing garden 1: drawing the garden
4	Setting up garden 2: blending soil	19	Designing garden 2: planting shrub
5	Setting up garden 3: fertilizing	20	Designing garden 3: planting herbs and bulbs
6	Setting up garden 4: making compost	21	Designing garden 4: planting vines
7	Plant propagation 1: seed propagation	22	Designing garden 5: mulching
8	Plant propagation 2: asexual reproduction	23	Reporting 1: monitoring the garden
9	Planting 1: tree planting	24	Reporting 2: making plant labels
10	Planting 2: potting plants	25	Pruning plants
11	Planting 3: transplanting	26	Getting rid of weeds
12	Event 1: enjoying herb tea	27	Pest control
13	Lecture: animals, birds, insects in garden	28	Event 4: yoga and picnic in the garden
14	Event 2: flower arrangement	29	Preparing garden for winter
15	Potting 1: repotting	30	Event 5: garden party

### 2.3 Measures

Baseline assessment was conducted before the therapeutic gardening program. After the final session, the post-test was administered. Demographic information on age, gender, marital status, education, and occupation was collected at the beginning of the program.

#### 2.3.1 Depressive symptoms

Depressive symptoms were assessed using the Mental Health Screening Tool for Depressive Disorders (MHS:D; Yoon et al., 2018). This 12-item inventory employs a five-point Likert scale. Higher scores indicate more severe symptoms over the past 2 weeks related to major depressive disorder. The MHS:D has demonstrated excellent internal consistency (Cronbach’s α offline version 0.943, online version 0.945) (Yoon et al., 2018).

#### 2.3.2 Anxiety symptoms

Anxiety symptoms were assessed using the Mental Health Screening Tool for Anxiety Disorders (MHS:A; Kim et al., 2021). The MHS:A is a self-report questionnaire with 11 items that are responded to on a five-point Likert scale. Higher scores indicate that more frequent anxiety symptoms were experienced over the past 2 weeks. The MHS:A has demonstrated excellent internal consistency (Cronbach’s *α* offline version 0.957, online version 0.956) (Kim et al., 2021).

#### 2.3.3 Perceived stress

The Korean version of the Perceived Stress Scale (K-PSS; Lee et al., 2012), initially developed by Cohen et al. (1983), was used to assess perceived stress. The K-PSS has demonstrated adequate internal consistency (Cronbach’s *α* = 0.82) (Lee et al., 2012). The K-PSS comprises 10 items that assess how individuals perceived and interpreted subjective stress experienced in the past month. Item responses range from 0 (never) to 4 (always). Higher scores indicate higher levels of stress. In the current study, scores below the average score of 20.69 were interpreted as below-average stress, while scores above 20.69 were interpreted as above-average stress (Lee et al., 2012).

#### 2.3.4 Mindful attention awareness

The Korean version of the Mindful Attention Awareness Scale (K-MAAS; Jeon et al., 2007), adapted from the MAAS (Brown and Ryan, 2003), is a 15-item inventory measuring mindful attention and awareness. Items are rated on a six-point scale with higher scores indicating greater mindfulness. The K-MAAS showed good internal consistency (Cronbach’s *α* = 0.87) (Jeon et al., 2007).

#### 2.3.5 Life satisfaction

The Korean version of the Satisfaction With Life Scale (K-SWLS; Cho and Cha, 1998), translated from the SWLS (Diener et al., 1985), was used to assess life satisfaction. The K-SWLS has five items rated on a seven-point scale (0 = completely disagree, 7 = completely agree). Higher scores indicate higher life satisfaction. The K-SWLS has demonstrated good internal consistency (Cronbach’s *α* = 0.85–0.90) (Cho and Cha, 1998).

### 2.4 Statistical analysis

All data analyses were conducted using Mplus version 8.3 (Muthén and Muthén, 2017) and SPSS version 27 (IBM Corp, 2020). Descriptive statistics and each measure’s reliability and validity were evaluated. To evaluate the validity, the Kaiser-Meyer-Olkin (KMO) coefficient, Bartlett test, item factor loadings, and average variance extracted (AVE) were used. The KMO coefficient and Bartlett’s test were used to examine sampling adequacy and suitability of the data for factor analysis (Burton and Mazerolle, 2011). KMO coefficients exceeding 0.8 indicate adequate levels, while coefficients below 0.5 indicate unacceptable levels (Kaiser and Rice, 1974). Regarding Bartlett’s test, the results should be significant for factor analysis to be suitable (Watson, 2017).

Confirmatory factor analysis (CFA) was conducted to compute factor loadings of each measure based on the factor structure of the original scales. The MHS:D, MHS:A, SWLS, and MAAS have one factor, and the PSS has two factors. The strict cutoffs for factor loadings are 0.32 (poor), 0.45 (fair), 0.55 (good), 0.63 (very good), and 0.71 (excellent) (Tabachnick et al., 2013). The cutoff for the AVE to satisfy convergent validity is 0.5 (Fornell and Larcker, 1981). Cronbach’s alpha was computed to examine the internal consistency reliability of each measure. A minimum acceptable alpha coefficient is 0.70 (Cortina, 1993; Tavakol and Dennick, 2011). The minimum composite reliability (CR) value in structural equation modeling (SEM) analysis is considered 0.70, according to Hair et al. (2021). Before SEM analysis, Pearson’s correlations were calculated to examine the association between each variable of two time points. To avoid Type I error, the Holm-Bonferroni method for multiple correlation analysis was used to adjust value of *p*s.

SEM with a two-wave autoregressive cross-lagged model was applied to explore the longitudinal mediation effects of mindfulness. The autoregressive cross-lagged model can be used to see how X affects Y through M over time by evaluating the lagged relationships from X to M, and from M to Y while controlling for the previous measurements of the same variables (Zhang and Yang, 2020). The mediation analysis with maximum likelihood estimation was conducted using 5,000 bootstrap samples to test indirect effects. Age and gender were included as covariates. Other demographic features were not added as covariates considering the modeling principle of parsimony (Yan et al., 2021; Kline, 2023). The mediation model fit was assessed using the root mean square error of approximation (RMSEA <0.06), comparative fit index (CFI > 0.95), and standardized root mean square residual (SRMR <0.08) according to the suggested cutoff criteria (Hu and Bentler, 1999). To handle missing values, the full information maximum likelihood method was employed.

## 3 Results

### 3.1 Participants’ characteristics

Table 2 presents the characteristics of the participants. Most participants were women (*n* = 204, 74%), and 45% were married (*n* = 125). Their mean age was 54.99 years (SD = 23.25). The participants were classified into the following categories based on their primary vulnerability: poor mental health conditions (*n* = 103, 37.3%), the elderly (*n* = 81, 29.3%), individuals with mild cognitive impairment and their caregivers (*n* = 71, 25.7%), and students with intellectual disabilities (*n* = 21, 7.6%). Nearly half of the participants reported no income source, including being students or unemployed (*n* = 128, 46.4%). Slightly more than half had completed upper high school (*n* = 146, *n* = 52.8%). Regarding clinical characteristics, one-fourth of the participants had been diagnosed with a mental disorder (*n* = 71, 25.7%).

**Table 2:** Baseline demographic characteristics of participants (*N* = 276).

Baseline characteristics	*N*	%
Gender		
Men	72	26.1
Women	204	73.9
Age, *M* (SD)	54.99 (23.25)	
Categorization of vulnerabilities		
Poor mental health conditions	103	37.3%
Elderly	81	29.3%
Mild cognitive impairment and caregivers	71	25.7%
Intellectual disability students	21	7.6%
Employment		
Unemployed	80	28.9
Student	48	17.5
Homemaker	46	16.7
Employed	56	20.3
Other	20	7.2
Unknown	26	9.4
Marital Status		
Never married	66	23.9
Married	125	45.3
Divorced	9	3.3
Widowed	42	15.2
Unknown	34	12.3
Education		
No Education	14	5.1
Elementary School (≤ 6 years)	41	14.9
Middle School (≤ 9 years)	40	14.5
High school (≤ 12 years)	79	28.6
University/College bachelor’s degree (≤ 16 years)	61	22.0
Higher Education (> 16 years)	6	2.2
Unknown	35	12.7
Mental disorder diagnosis		
None	205	74.3
Yes	71	25.7

*M* and SD are used to represent mean and standard deviation, respectively.

### 3.2 Validity and reliability of the measures

Table 3 shows each measure’s reliability and construct validity. All factor loading estimates were over 0.45 except for Item 7 on the K-PSS and Item 13 on the K-MAAS. The KMO coefficients of the MHS:D, MHS:A, K-SWLS, and K-MAAS were above 0.8, indicating adequate levels, and the coefficient for the K-PSS was 0.77, indicating an acceptable level. The results of Bartlett’s test were significant for all measures. The AVE values of the MHS:D, MHS:A, and K-SWLS were over 0.5. The AVE values of the K-MAAS and K-PSS did not reach 0.5, but were close to it. All Cronbach’s alpha coefficients and CR values exceeded 0.70, affirming the measures’ reliability.

**Table 3:** Test of construct validity and reliability of each measurement model.

Measures	Items		Standardized factor loadings	KMO	Bartlett’s test	AVE	Cronbach’s alpha	C.R.
MHS:D	1		0.767	0.930	0.000	0.507	0.912	0.917
	2		0.694					
	3		0.681					
	4		0.684					
	5		0.785					
	6		0.618					
	7		0.808					
	8		0.828					
	9		0.793					
	10/11		0.570					
	12		0.531					
MHS:A	1		0.787	0.927	0.000	0.541	0.927	0.928
	2		0.757					
	3		0.744					
	4		0.651					
	5		0.780					
	6		0.772					
	7		0.679					
	8		0.647					
	9		0.714					
	10		0.755					
	11		0.784					
K-SWLS	1		0.656	0.839	0.000	0.581	0.866	0.871
	2		0.816					
	3		0.924					
	4		0.768					
	5		0.604					
K-MAAS	1		0.501	0.909	0.000	0.449	0.919	0.923
	2		0.682					
	3		0.687					
	4		0.739					
	5		0.744					
	6		0.478					
	7		0.709					
	8		0.722					
	9		0.791					
	10		0.750					
	11		0.551					
	12		0.724					
	13		0.441					
	14		0.758					
	15		0.645					
K-PSS	F1	1	0.702	0.766	0.000	0.408	0.702	0.870
		2	0.730					
		3	0.638					
		6	0.590					
		9	0.594					
		10	0.727					
	F2	4	0.688					
		5	0.695					
		7	0.365					
		8	0.575					

MHS:D, mental health screening tool for depressive disorders; MHS:A, mental health screening tool for anxiety disorders; K-SWLS, Korean version of the satisfaction with life scale; K-MAAS, Korean version of the mindful attention awareness scale; K-PSS, Korean version of the perceived stress scale; KMO, Kaiser-Mayer-Orkin; AVE, average variance extraction; C.R., composite reliability.

### 3.3 Associations between mental health variables

Table 4 presents the means, standard deviations, and correlations for the two time points. The mean scores for depression, anxiety, and stress decreased from TI to T2, and life satisfaction and mindfulness scores increased from TI to T2. The variables were significantly correlated with each other, except for the associations between mindfulness (T1) and depression (T2), mindfulness (T1) and life satisfaction (T2), and stress (T2) and all other variables (T1).

**Table 4:** Means, standard deviations, and correlations with confidence intervals of mental health variables.

Variable	*M*	SD	1	2	3	4	5	6	7	8	9
1. Depression_T1	11.69	10.01									
2. Depression_T2	6.81	8.61	0.37**								
			[0.26, 0.47]								
3 Anxiety_T1	11.72	9.73	0.82**	0.37**							
			[0.78, 0.86]	[0.27, 0.47]							
4. Anxiety_T2	6.83	8.79	0.31**	0.87**	0.36**						
			[0.20, 0.42]	[0.83, 0.89]	[0.25, 0.46]						
5. Life Satisfaction_T1	20.29	6.75	−0.45**	−0.19**	−0.44**	−0.16*					
			[−0.54, −0.35]	[−0.30, −0.07]	[−0.53, −0.34]	[−0.27, −0.04]					
6. Life Satisfaction_T2	24.08	6.89	−0.25**	−0.44**	−0.25**	−0.46**	0.42**				
			[−0.36, −0.14]	[−0.53, −0.33]	[−0.36, −0.14]	[−0.55, −0.36]	[0.32, 0.51]				
7. Mindfulness_T1	61.62	15.36	−0.41**	−0.13	−0.45**	−0.15*	0.41**	−0.00			
			[−0.51, −0.29]	[−0.25, 0.00]	[−0.54, −0.34]	[−0.27, −0.02]	[0.30, 0.51]	[−0.13, 0.13]			
8. Mindfulness_T2	69.36	15.16	−0.33**	−0.48**	−0.35**	−0.54**	0.23**	0.51**	0.42**		
			[−0.44, −0.22]	[−0.57, −0.37]	[−0.46, −0.23]	[−0.63, −0.44]	[0.10, 0.34]	[0.40, 0.60]	[0.31, 0.52]		
9. Stress_T1	18.22	5.22	0.59**	0.24**	0.62**	0.25**	−0.42**	−0.25**	−0.45**	−0.32**	
			[0.50, 0.67]	[0.11, 0.35]	[0.54, 0.69]	[0.12, 0.36]	[−0.52, −0.30]	[−0.36, −0.12]	[−0.54, −0.34]	[−0.43, −0.20]	
10. Stress_T2	14.42	6.73	0.03	0.56**	0.07	0.62**	−0.12	−0.53**	0.01	−0.55**	0.26**
			[−0.10, 0.16]	[0.46, 0.64]	[−0.06, 0.19]	[0.54, 0.70]	[−0.25, 0.00]	[−0.61, −0.43]	[−0.12, 0.13]	[−0.63, −0.45]	[0.14, 0.38]

*M* and SD are used to represent mean and standard deviation, respectively. Values in square brackets indicate the 95% confidence interval for each correlation. *indicates *p* < 0.05. **indicates *p* < 0.01.

### 3.4 Autoregressive cross-lagged mediation analysis

*H1:* Mindfulness (T2) mediates the relationship between depression (T1) and life satisfaction (T2).

**Figure 1:**
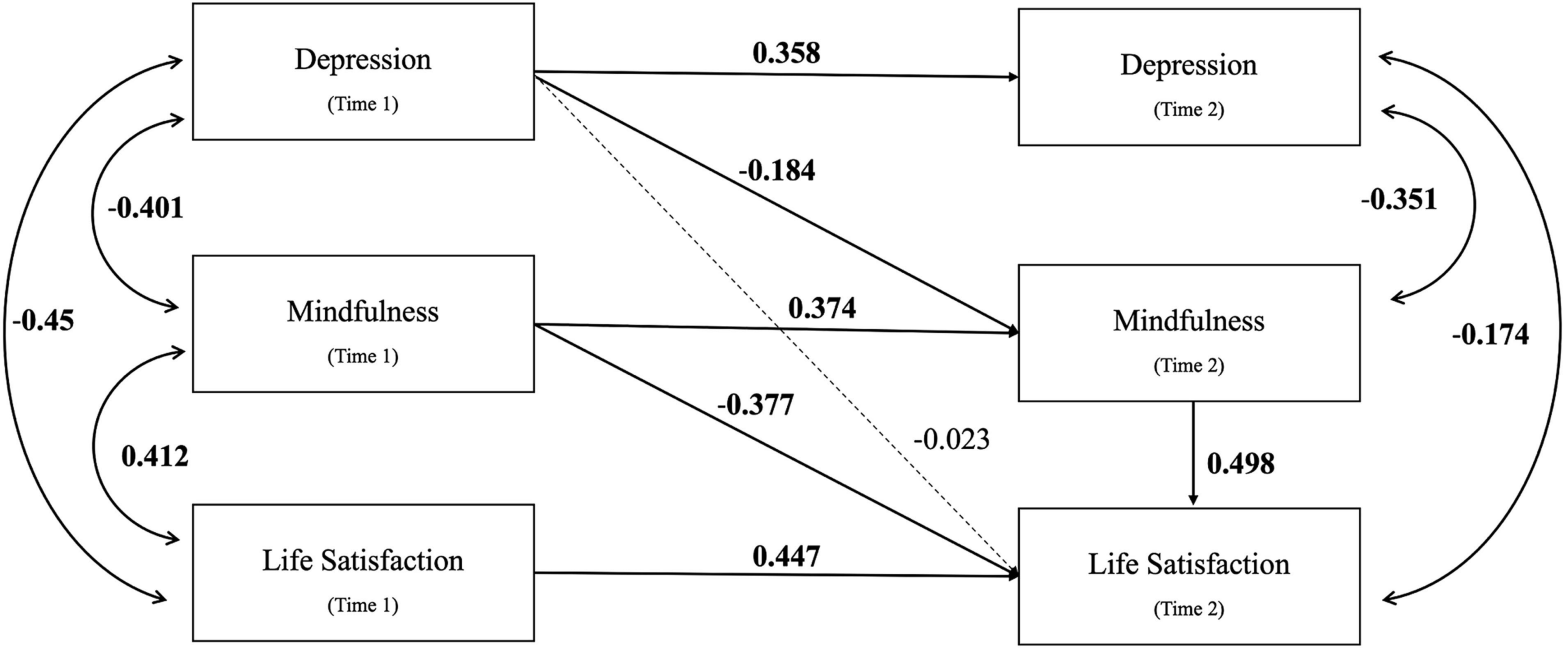
The two-wave cross-lagged mediation model for Hypothesis 1. The figure shows the standardized regression coefficients of all paths. Solid and dashed lines indicate significant and non-significant paths, respectively. Time 1-Pre-intervention; Time 2-Post-intervention.

Figure 1 illustrates the relationships among depression, mindfulness, and life satisfaction. The model fit indices of the autoregressive cross-lagged models are provided in Table 5. The mediation model had an excellent fit to the data (*χ*^2^ = 8.348, df = 9, *p* = 0.50; RMSEA = 0.000; CFI = 1.00; SRMR = 0.032). The autoregressive effects of the variables at T1 to T2 were significant (p<0.001), as shown in Table 6. Longitudinal mediation analysis revealed that the indirect effect of depression (T1) on life satisfaction (T2) through mindfulness (T2) was significant [*β* = −0.092, 95% CI (−0.166,-0.018), *p* = 0.015]. Both the direct and total effects were not statistically significant, indicating the significant mediating role of mindfulness.

**Table 5:** The model fit indices of the hypothesized autoregressive cross-lagged mediation model.

Hypothesis	Mediating Path	$x^{2}$(df)	Value of *p* of $x^{2}$ test	RMSEA	CFI	SRMR
H1	DEP (T1) → MF (T2) → LS (T2)	8.348 (9)	0.4995	0.000	1.000	0.032
H2	ANX (T1) → MF (T2) → LS (T2)	13.497 (9)	0.142	0.042	0.988	0.038
H3	ST (T1) → MF (T2) → LS (T2)	23.092 (9)	0.006	0.075	0.963	0.051

DEP, depression; ANX, anxiety; ST, stress; MF, mindfulness; LS, life satisfaction.

**Table 6:** Mediation model parameters for each hypothesis.

Hypothesis	Path			Standardized estimate	S.E.	95% CI		Value of *p*
						Lower	Upper	
H1	Mediating effects	DEP (T1) → MF (T2) → LS (T2)	Total	−0.114	0.066	−0.243	0.014	0.081
			Indirect	−0.092	0.038	−0.166	−0.018	0.015*
			Direct	−0.023	0.057	−0.134	0.089	0.692
	Autoregressive effects	DEP (T1) → DEP (T2)		0.358	0.071	0.219	0.496	< 0.001***
		MF (T1) → MF (T2)		0.374	0.059	0.257	0.490	< 0.001***
		LS (T1) → LS (T2)		0.447	0.059	0.332	0.562	< 0.001***
	Predictive effects	DEP (T1) → MF (T2)		−0.184	0.065	−0.352	−0.057	0.005**
		MF (T1) → LS (T2)		−0.377	0.062	−0.499	−0.255	< 0.001***
H2	Mediating effects	ANX (T1) → MF (T2) → LS (T2)	Total	−0.123	0.063	−0.247	0.001	0.052
			Indirect	−0.088	0.039	−0.164	−0.013	0.022*
			Direct	−0.035	0.059	−0.150	0.081	0.556
	Autoregressive effects	ANX (T1) → ANX (T2)		0.344	0.064	0.218	0.469	< 0.001***
		MF (T1) → MF (T2)		0.361	0.058	0.248	0.475	< 0.001***
		LS (T1) → LS (T2)		0.455	0.056	0.345	0.565	< 0.001***
	Predictive effects	ANX (T1) → MF (T2)		−0.176	0.069	−0.311	−0.042	0.010*
		MF (T1) → LS (T2)		−0.395	0.062	−0.515	−0.274	< 0.001***
H3	Mediating effects	ST (T1) → MF (T2) → LS (T2)	Total	−0.087	0.064	−0.213	0.039	0.174
			Indirect	−0.037	0.035	−0.107	0.032	0.294
			Direct	−0.050	0.059	−0.166	0.066	0.399
	Autoregressive effects	ST (T1) → ST (T2)		0.222	0.063	0.097	0.346	< 0.001***
		MF (T1) → MF (T2)		0.444	0.050	0.346	0.541	< 0.001***
		LS (T1) → LS (T2)		0.438	0.057	0.326	0.550	< 0.001***
	Predictive effects	ST (T1) → MF (T2)		−0.072	0.065	−0.200	0.056	0.271
		MF (T1) → LS (T2)		−0.381	0.068	−0.514	−0.248	< 0.001***

*indicates *p* < 0.05. **indicates *p* < 0.01. ***indicates *p* < 0.001; DEP, depression; ANX, anxiety; ST, stress; MF, mindfulness; LS, life satisfaction.

*H2:* Mindfulness (T2) mediates the relationship between anxiety (T1) and life satisfaction (T2).

**Figure 2:**
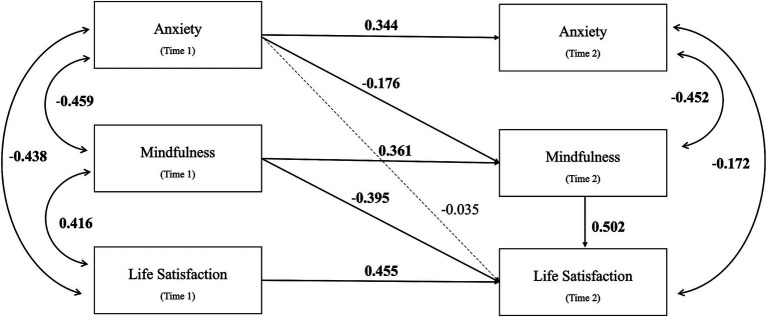
The two-wave cross-lagged mediation model for Hypothesis 2. The figure shows the standardized regression coefficients of all paths. Solid and dashed lines indicate significant and non-significant paths, respectively. Time 1-Pre-intervention; Time 2-Post-intervention.

Figure 2 illustrates the relationships among anxiety, mindfulness, and life satisfaction. The mediation model had a good fit to the data (*χ*^2^ = 13.479, df = 9, *p* = 0.142; RMSEA = 0.042; CFI = 0.988; SRMR = 0.038) (Table 5). The autoregressive effects of the variables at T1 to T2 were significant (p<0.001) (Table 6). The mediating analysis indicated that the indirect effect of anxiety (T1) on life satisfaction (T2) through mindfulness (T2) was significant [*β* = −0.088, 95% CI (−0.164,-0.013), *p* = 0.022]. Moreover, the total and direct effects showed no significant effect, indicating that the relationship between anxiety and life satisfaction was primarily explained through the mediating effect of mindfulness.

*H3:* Mindfulness (T2) mediates the relationship between stress (T1) and life satisfaction (T2).

**Figure 3:**
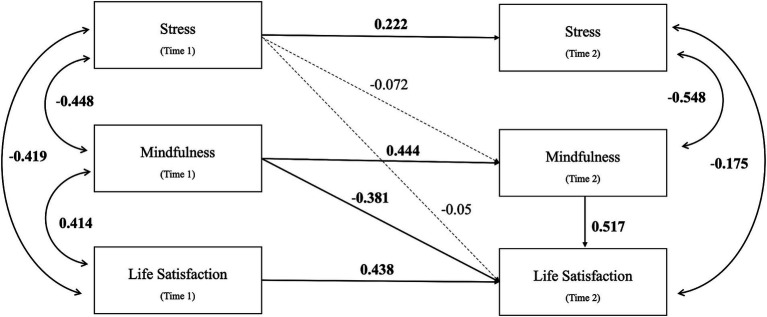
The two-wave cross-lagged mediation model for Hypothesis 3. The figure shows the standardized regression coefficients of all paths. Solid and dashed lines indicate significant and non-significant paths, respectively. Time 1-Pre-intervention; Time 2-Post-intervention.

Figure 3 illustrates the relationships among stress, mindfulness, and life satisfaction. The mediation model had a fair fit to the data (*χ*^2^ = 23.092, df = 9, *p* = 0.006; RMSEA = 0.075; CFI = 0.963; SRMR = 0.051) (Table 5). The autoregressive effects of the variables at T1 to T2 were significant (*p* < 0.001) (Table 6). However, there was no statistically significant mediating effect of mindfulness (T2) on stress (T1) and life satisfaction (T2) [*β* = −0.037, 95% CI (−0.107, 0.032), *p* = 0.294].

## 4 Discussion

### 4.1 Main findings

This study evaluated the possible mechanism of NBT by examining the relationships between life satisfaction and depression, anxiety, and stress with mindfulness as a mediator in a two-wave autoregressive mediation model. In general, significant improvements were observed in the mean scores of all psychological variables from pre- to post-intervention, which aligns with previous studies showing that NBT had a positive impact on the psychological well-being of participants (Han et al., 2018; Kim and Park, 2018; Chan et al., 2022; Yang et al., 2022; Hyvönen et al., 2023).

The mediation model was confirmed either in whole or in part. Mindfulness (T2) mediated the path from depression (T1) and anxiety (T1) to life satisfaction (T2). Many studies have demonstrated that both nature and mindfulness-based therapy are effective in reducing symptoms of anxiety and depression in diverse populations (Maas et al., 2009; Hofmann et al., 2010; Jedel et al., 2013; Beyer et al., 2014; Spinhoven et al., 2022). Additionally, Korpela et al. (2014) analyzed data from 3,060 Finnish participants and discovered that the relationship between nature-based recreation and emotional well-being was mediated by restorative experiences. A plausible explanation for this result might be that mindfulness, which enables participants to be fully present in the moment, fostering nonjudgmental acceptance, may have contributed to helping individuals alleviate distress and cultivate more accepting responses to unpleasant thoughts and emotions. This, in turn, can positively impact one’s quality of life (Baer, 2003; Azad Marzabadi et al., 2021). Mindfulness also strengthens self-regulation (Bishop et al., 2004), which leads to enhanced resilience, and subsequently, increased psychological well-being (Duan, 2016). Although there are relatively few nature-based mindfulness interventions, their positive effects seem promising (Djernis et al., 2021; Owens and Bunce, 2022). For instance, a randomized controlled trial exploring the effects of nature-based mediation on rumination, depressive symptoms, and well-being in youths revealed a significant decrease in depressive rumination and increased well-being during follow-up in the nature condition compared with an indoor meditation group and an active control group (Owens and Bunce, 2022). Furthermore, Vitagliano et al. (2023) proposed nature-based mindfulness training for anxious college students, arguing for the potential benefits of combining mindfulness practices and nature exposure as a promising therapeutic approach for sustainable anxiety management. A study comparing the effectiveness of mindfulness-based stress reduction conducted indoors, outdoors, and in natural environments found that nature-based stress reduction resulted in more connections with nature and enhanced reflection, supporting the potential value of nature as a therapeutic resource (Choe et al., 2020).

Although the mean stress score decreased at post-intervention compared to pre-intervention, mindfulness (T2) did not mediate the relationship between stress (T1) and life satisfaction (T2). While Djernis et al. (2021) did not find a significant effect on perceived stress after implementing their mindfulness intervention, most studies investigating nature and mindfulness-based programs found significant effects in reducing stress and improving well-being (Grossman et al., 2004; Stigsdotter et al., 2018; Yao et al., 2021). One possible explanation for this inconsistent result is that there are other variables or mediators that can impact the relationship between stress and life satisfaction, which were not considered in our study. For example, coping strategies, emotional regulation, or self-efficacy could have contributed to the stress–life satisfaction relationship (Lee et al., 2016; Gori et al., 2020; Xu et al., 2021). One study examining the possible mediators in the relationship between green areas and the well-being of inhabitants revealed that among physical activity, perceived stress, social cohesion, concentration ability, and neighborhood satisfaction, only neighborhood satisfaction was a significant mediator (Van Herzele and de Vries, 2012). Additionally, the relationship between mindfulness and stress can be complex and influenced by multiple factors such as the type of intervention employed, duration, intensity, and/or population studied (Christopher et al., 2009; Davidson and Kaszniak, 2015; Igartua and Hayes, 2021). Another possibility might be that the participants in the present study did not have high levels of stress before the intervention, contributing to a floor effect, which makes it difficult to detect mediation effects owing to insufficient variability in the levels of stress. Further research is needed to explore the potential reasons why mindfulness may or may not mediate the relationship between stress and life satisfaction. Nevertheless, these findings contribute to our understanding of the mechanisms underlying the effects of nature-based mindfulness interventions on psychological well-being and highlight the differential impact of mindfulness on various psychological factors.

### 4.2 Limitations

This study has several limitations that warrant consideration. First, the therapeutic gardening intervention was not specifically designed to incorporate active mindfulness elements, despite encompassing the essence of mindfulness through interaction with nature. Although this could have potentially influenced the results, the significant findings support the notion that elements of mindfulness exist in NBT (Cimprich and Ronis, 2003; Howell et al., 2011; Van Gordon et al., 2018). Randomized controlled trials of NBT incorporating mindfulness as a key component should be tested in future studies to confirm our findings (Vitagliano et al., 2023). Second, while the longitudinal mediation design strengthens evidence for causal relationships compared to cross-sectional designs (Little, 2013), the use of only two time points may have constrained the ability to capture more nuanced temporal relationships and could not ascertain complete mediation (Hamaker et al., 2015). Including additional time points could provide a comprehensive understanding of the mediation effects. Third, self-report questionnaires may be affected by response bias; therefore, future studies should use objective measures and replicate the study with different populations. Finally, while gender and age were accounted for, other demographic characteristics like marital status, mental disorder diagnosis and education level were not controlled, as preliminary analyses indicated minimal impact and in pursuit for a parsimonious model. Also, the heterogeneity of our sample across various categories of vulnerability was not explicitly addressed in the analysis. These aspects, however, may have contributed to the observed outcomes. Future studies should use robust control measures and subgroup analyses to investigate the influence of demographic factors and diverse populations.

## 5 Conclusion

This study represents a pioneering effort to explore the mediation effects of mindfulness within the context of NBT, highlighting the importance of considering the temporal dynamics and mechanisms involved in the relationship between NBT and psychological well-being. Mindfulness mediated the relationship between depression and anxiety at T1 and life satisfaction at T2, thus highlighting the importance of cultivating mindfulness as a core component in nature-based interventions to enhance their effectiveness in improving mental health outcomes.

## Data availability statement

The original contributions presented in the study are included in the article/supplementary material, further inquiries can be directed to the corresponding author.

## Ethics statement

The studies involving humans were approved by the Korea University Institutional Review Board (protocol no. KUIRB-2022-0218-03, 05/04/2022). The studies were conducted in accordance with the local legislation and institutional requirements. All participants gave written informed consent, and for those under 18 years old, written consent was obtained from their legal guardians/next of kin.

## Author contributions

MK: Writing – original draft, Writing – review & editing, Conceptualization, Data curation, Formal analysis, Investigation, Methodology. YY: Data curation, Formal analysis, Writing – original draft, Investigation, Methodology. HK: Formal analysis, Methodology, Writing – original draft, Investigation. SJ: Writing – review & editing, Resources. H-YJ: Writing – review & editing, Resources. K-HC: Conceptualization, Project administration, Supervision, Writing – review & editing, Funding acquisition.
